# Gut eosinophils and their impact on the mucus‐resident microbiota

**DOI:** 10.1111/imm.13110

**Published:** 2019-09-17

**Authors:** Gurdeep Singh, Andrew Brass, Christopher G. Knight, Sheena M. Cruickshank

**Affiliations:** ^1^ Faculty of Biology, Medicine and Health Lydia Becker Institute of Immunology and Inflammation Manchester Academic Health Science Centre The University of Manchester Manchester UK; ^2^ Faculty of Biology, Medicine and Health Division of Informatics, Imaging and Data Sciences The University of Manchester Manchester UK; ^3^ Faculty of Science and Engineering School of Natural Sciences The University of Manchester Manchester UK

**Keywords:** co‐housing, diversity, gut barrier, littermate control, plasma cells

## Abstract

The gut has the largest commensal bacterial population in the body and its composition can be impacted by host factors such as production of immunoglobulin A (IgA). Eosinophils in the gut have been implicated in the production of antibacterial factors and maintenance of IgA‐secreting plasma cells. We used an eosinophil‐deficient mouse (*∆dblGATA‐1* ^*−/−*^) and littermate controls to investigate the role of eosinophils in the regulation of the microbiota, with particular emphasis on mucus‐resident species in the small and large intestine. We found no differences in IgA production or IgA‐expressing plasma cells between naive littermates in the small or large intestine. However, denaturing gel gradient electrophoresis revealed differences in the bacterial communities of the mucus and stools between wild‐type mice and *∆dblGATA‐1* ^*−/−*^ mice, with the greatest separation between the mucus microbial communities. Mucus‐resident bacteria in *∆dblGATA‐1* ^*−/−*^ mice had reduced diversity in the mucus compared with the stools. A quantitative PCR panel of selected bacteria showed that the most significant differences in the microbiota were between mucus‐resident bacteria and those in stool, such as the abundance of Clostridiales and Bacteroides. Our data implicate eosinophils in the regulation of the microbiota, especially the bacteria most hyperlocal to the gut barrier. Although we see differences between host genotypes in the overall microbial communities, further work is required to establish specifically which bacteria are different between these groups. Most importantly, the data revealed that the mucus and stool microbiota are discrete communities. Stool analysis alone may be insufficient to comprehensively explore and define the role of the gut microbiota in health and disease.

**Abbreviations**
DGGE: denaturing gel gradient electrophoresis
Het: heterozygous
IgA: immunoglobulin A
PBS: phosphate‐buffered saline
qPCR: quantitative polymerase chain reaction
WT: wild‐type

## Introduction

An effective gut barrier is crucial for our health by helping prevent entry of pathogens while enabling the entry of helpful nutrients and commensal bacterial products. The gut barrier comprises a mucus layer that is situated on top of a monolayer of intestinal epithelial cells. Intestinal epithelial cells and immune cells secrete and transport an array of antimicrobial factors that include antibacterial peptides and secreted immunoglobulin A (IgA), which are particularly concentrated within the mucus layer.^1^ Another crucial component of the gut barrier is the gut microbiota, which also serves to protect against pathogens by competing for resources or production of anti‐microbial factors.^2^ A variety of host cells are involved in the maintenance of the gut barrier with immune cells such as eosinophils thought to play a key role.^3^

Eosinophils are resident immune cells found all along the length of the gut with the highest proportion in the small intestine.^4^ Eosinophils have been linked to the regulation of IgA‐secreting plasma cells and the gut mucus layer,^5^ both major components of the gut barrier. Additionally, eosinophils have been linked to the maintenance of intestinal permeability.^3^ Therefore, understanding how eosinophils contribute to barrier integrity and their impact on the gut microbiota is an important area of investigation.

The microbiota varies along the length of the gastrointestinal tract with fewer bacteria found at the top of the small intestine and the greatest number and most varied communities found in the large intestine.^6^ The gut microbiota then exists within two major niches within the gut, the lumen and the gut mucus layer. Given these distinctions among gut microbial communities, it is perhaps surprising that much of the work to date has focused on characterizing stool bacteria that are unlikely to fully recapitulate this diversity. The importance of investigating different microbial niches is crucial, as it is likely that the microbiota within these niches have different functional impacts for the host. Indeed, our previous work has demonstrated that the colonic mucus microbiota, but not the stool bacteria, changes before the onset of colitis.^7^

Here, we investigate how eosinophil deficiency impacts upon both IgA‐secreting plasma cells and the gut microbiota, with a particular focus on exploring small versus large intestine and mucus versus stools. Using littermate‐controlled wild‐type (WT) and eosinophil‐deficient (*∆dblGATA‐1* ^*−/−*^) mice, we saw no differences in the numbers of IgA‐secreting plasma cells between genotypes. Furthermore, broad analysis of the stool and mucus microbiota revealed significant differences between strains, with stronger separation in our mucus samples. However, specific analysis of which types of bacteria were different indicated that the microbial niche (i.e. stool versus mucus) was the most powerful influencing factor on the gut microbiota.

## Materials and methods

#### Animal maintenance

*ΔdblGATA‐1**^-/-^*** mice^8^ (kindly provided by Professor Avery August, Pennsylvania State University, PA) on a C57BL/6 background were crossed with WT C57BL/6 mice to produce the F_2_ generation, which were used for all experiments. WT male mice and heterozygous (Het) female mice, and *ΔdblGATA‐1* ^*−/−*^ mice from the same litters were used for all subsequent experiments. It should be noted that due to the nature of the mutation, female mice could only be Het in this study but function as WTs, given the presence of eosinophils in these mice (see Supplementary material, Fig. S1). Food (Beekay Rat and Mouse Diet No. 1 pellets; B&K Universal, Hull, UK) and water were available *ad libitum*. Ambient temperature was maintained at 21° (± 2°) and the relative humidity was 55% (± 10%) with a 12 hr/12 hr light/dark cycle. Male mice at 12 weeks old and female mice at 12 weeks and 1 year of age were used for experiments. All animals were kept under specific, pathogen‐free conditions at the University of Manchester and experiments were performed according to the regulations issued by the Home Office under amended ASPA, 2012.

#### Physiological parameters

A glucose tolerance test was performed in female, young and aged Het and *ΔdblGATA‐1* ^*−/−*^ mice and systolic blood pressure and heart rate were measured as described previously.^9^

#### Sample preparation

Faecal samples were collected into sterile Eppendorf tubes and snap‐frozen on dry ice. Mice were then killed by CO_2_ inhalation. Small intestinal and distal colon snips were fixed in either Carnoy's fixative (60% ethanol absolute, 30% chloroform and 10% glacial acetic acid) to preserve the mucus or KP‐CryoCompound (VWR, Lutterworth, UK). The remaining colon was opened up and any remaining faecal matter was removed and gently washed away with phosphate‐buffered saline (PBS; Sigma, Poole, UK). The inner surface of the colon was scraped using a cell scraper and InhibitEX buffer (Qiagen, Manchester, UK) to remove mucus from the mucus lining, which was then snap‐frozen. Serum was incubated at 37° for 2 hr, before centrifugation at 7000 *g* for 10 min to collect the supernatant. The supernatant was stored at −80°.

#### Histology and staining

Carnoy's fixed samples were incubated in two changes of dry methanol (Sigma) for 30 min each, followed by absolute ethanol (ThermoFisher Scientific, Crawley, UK) for two incubations at 30 min each. Tissue cassettes were processed in a Micro‐spin Tissue Processor STP120 (ThermoFisher Scientific) and immersed in paraffin using a Leica Biosystems embedding station (Leica Biosystems, Milton Keynes, UK), with the luminal surface of the colon exposed for tissue sectioning. Tissue sections (5 μm) were cut using a Leica Biosystems microtome and adhered to uncoated microscope slides (ThermoFisher Scientific). Slides were dried for 48 hr at 50° before use. Haematoxylin & eosin and goblet cell staining were performed and analysed as described previously.^7^

#### Immunostaining for eosinophils

Frozen sections were dried at room temperature and fixed in cold 4% paraformaldehyde (Sigma) in dH_2_O at −20° for 5 min. Slides were rehydrated in wash buffer, 0·05% bovine serum albumin (BSA) (Sigma) in PBS, for 5 min. Slides were blocked with 5% BSA in PBS, washed in PBS and avidin/biotin activity was blocked according to manufacturer's instructions with the Avidin/Biotin Blocking System (BioLegend, London, UK). Slides were stained and co‐stained with biotinylated anti‐Siglec F antibody (Bio‐Techne, Abingdon, UK) and fluorescein isothiocyanate‐conjugated cytokeratin antibody (Sigma). Sections were then stained with Streptavidin‐horseradish peroxidase solution (Vector Laboratories, Peterborough, UK), washed with PBS and stained with tyramide‐Cy3 (Perkin Elmer, Seer Green, UK) before being left to air dry. Slides were mounted with VECTASHIELD® HardSet Antifade Mounting Medium with DAPI (Vector Laboratories) and imaged with a Nikon ECLIPSE Ci‐L microscope with a DS‐Fi3 Microscope Camera (Nikon, Kingston upon Thames, UK). Red cells were positive for Siglec F (eosinophils) and green cells were positive for cytokeratin (epithelial cells). Co‐localized staining (i.e. yellow cells) were discarded as tuft cells.

#### Fluorescence *in situ* hybridization

Fluorescence in situ hybridization (FISH) was performed as described previously.^7^ In brief, FISH staining was performed using the universal bacterial probe‐EUB338 (5′‐Cy3‐GCTGCCTCCCGTAGGAGT‐3′), followed by immunostaining with a rabbit polyclonal MUC2 antibody and goat anti‐rabbit Alexa‐Fluor 488 antibody (Life Technologies, Paisley, UK). The thickness of the inner mucus barrier was quantified by measuring the distance between the epithelium and the outer mucus layer. Bacterial penetrance was also investigated by assigning values from 0 to 4 depending on where bacteria were localized: 0 = bacteria in the lumen and outer mucus layer, 1 = bacteria in the inner mucus layer, 2 = bacteria in contact with the epithelium, 3 = bacteria in the crypts, 4 = bacteria in the lamina propria. All slides were scored in a blind manner.

#### Flow cytometry for IgA plasma cells

Whole colon and small intestine were harvested and fat was removed from the tissue and washed thoroughly in PBS. Tissue was placed into warm strip buffer (PBS containing 1% fetal bovine serum, 0·5 m ethylenediamine tetraacetic acid and 0·2 mm dithiothreitol (all Sigma), and incubated for 10 min at 37° on a shaking incubator (Cole Palmer, St Neots, UK) at 205 r.p.m. The supernatant was removed, and the process was repeated before incubation in digest buffer (RPMI‐1640, 10% fetal calf serum, 1% penicillin‐streptomycin, 0·025 mg/ml deoxyribonuclease I from bovine pancreas (all Sigma) and 0·01% collagenase‐dispase (Roche Diagnostics Ltd, Burgess Hill, UK) for 45 min at 37° on a shaking incubator at 205 r.p.m. Cells were strained through a 70‐μm sieve and pelleted at 450 *g* for 5 min. Cells were resuspended in FACS buffer (0·5% BSA in PBS) and stained with B220‐allophycocyanin‐Cy7, CD3‐Peridinin chlorophyll protein, CD19‐AmCyan, MHCII‐PacBlue (ThermoFisher Scientific), IgA‐phycoerythrin (eBioscience‐ThermoFisher Scientific) and CD45‐BV650 (BioLegend, San Diego, CA). After staining, cells were fixed in IC Fixation Buffer (ThermoFisher Scientific), resuspended in FACS buffer and then analysed using a BD Biosciences LSR Fortessa (BD Biosciences, Oxford, UK). Doublets and dead cells were excluded from analysis. IgA^+^ plasma cells were reported as a proportion of total live CD45^+^ cells. IgA^+^ plasma cells were gated as MHCII^+^, CD3^−^, IgA^+^ and B220^−^.

#### IgA ELISA

An enzyme‐linked immunosorbent assay (ELISA) was performed using the Invitrogen™ eBioscience™ Mouse IgA ELISA Ready‐SET‐Go!™ Kit (ThermoFisher Scientific, Crawley, UK), according to the manufacturer's instructions. The ELISA was read using a VersaMax microplate reader (Molecular Devices, San Jose, CA).

#### 16S DNA extraction, amplification and purification

DNA extraction was performed using a Qiamp® Fast Stool DNA Mini Kit (Qiagen), using a modified version of the manufacturer's instructions. Faecal samples were incubated in Inhibitex buffer (Qiagen) and mechanically disaggregated, before incubation at 95° for 30 min. Mucus samples were centrifuged (13 000 *g* for 10 min) and the mucus pellets were incubated in Inhibitex buffer, mechanically disaggregated and incubated at 95° for 30 min. Three hundred microlitres of the resulting lysate was used for the subsequent steps, which were then performed according to the manufacturer's instructions. Optical density at 260 nm was recorded using a UV1101 spectrophotometer (Biochrom Ltd, Cambridge, UK) to measure DNA concentration. For the identification of different bacterial species, the 16S rRNA gene was amplified using the universal primer pairs P3_GC‐341F and P2_518 (Table 1), as described previously.^7^

**Table 1:** List of primers used

Gene	Primers	Sequence (5′‐3′)
GATA‐1	GATA 1 S	CCAATCCTCTGGACTCCCA
	GATA 1 AS	CCTACTGTGTACCAGGCTAT
16S rRNA	P3_GC‐341F	CGCCCGCCGCGCGCGGCGGGCGGGGCG GGGGCACGGGGGGCCTACGGGAGGCAGCAG
	P2_518R	ATTACCGCGGCTGCTGG
Universal	UniF340	ACTCCTACGGGAGGCAGCAGT
	UniR514	ATTACCGCGGCTGCTGGC
Actinobacteria	Act664F	TGTAGCGGTGGAATGCGC
	Act941R	AATTAAGCCACATGCTCCGCT
Bacteroidetes	Bac960F	GTTTAATTCGATGATACGCGAG
	Bac1100R	TTAASCCGACACCTCACGG
Deferribacteres	Defer1115F	CTATTTCCAGTTGCTAACGG
	Defer1265R	GAGHTGCTTCCCTCTGATTATG
Firmicutes	Firm934F	GGAGYATGTGGTTTAATTCGAAGCA
	Firm934R	AGCTGACGACAACCATGCAC
Verrucomicrobia	Ver1165F	TCAKGTCAGTATGGCCCTTAT
	Ver1263R	GAGHTGCTTCCCTCTGATTATG
Bacteroides	BactF285	GGTTCTGAGAGGAGGTCCC
	UniR338	GCCTCAAGGGCACAACCTCCAAG
Clostridiales	UniF338	ACTCCTACGGGAGGCAGC
	C.cocR491	GCTTCTTAGTCAGGTACCGTCAT
*Enterobacteriaceae*	Uni515F	GTGCCAGCAGCCGCGGTAA
	Ent826R	GCCTCAAGGGCACAACCTCCAAG
*Lachnospiraceae*/*Rumminococceae*	LachnoRumF	CGGTACCTGACTAAGAAGC
	LachnoRumR	AGTTTCATTCTTGCGAACG
*Akkermansia muciniphila*	Amuc_1599F	GACCGGCATGTTCAAGCAGACT
	Amuc_1599R	AAGCCGCATTGGGATTATTTGTT
Segmented filamentous bacteria	SFB736F	GACGCTGAGGCATGAGAGCAT
	SFB844R	GACGGCACGGATTGTTATTCA

#### Denaturing gradient gel electrophoresis

A denaturing gradient gel was prepared according to the methods initially developed by Fischer and Lerman.^10^ The gel was run as described previously.^7^ The gel was then analysed using phoretix Software (TOTALLAB, Newcastle upon Tyne, UK). Lane boundaries were defined to correct for any potential distortions during the gel run and manually curated to ensure that the bands detected were not artefacts. Reference bands were selected to align bands across the gel and ‘Rf values’ were generated to measure the bands migration. A binary matrix was then generated based on the Rf values, with 0s and 1s indicating the absence or the presence of a bacterial ‘species’ in a sample. This matrix was processed in R.^11^ Specifically, the ‘ecodist’ package^12^ was used to generate a Bray Curtis Dissimilarity matrix from the binary matrix to compare the presence or absence of species (bands) between groups, i.e. WT lumen, WT mucus, *ΔdblGATA‐1* ^*−/−*^ lumen and *ΔdblGATA‐1* ^*−/−*^ mucus. The same packages were used to perform non‐metric multidimensional scaling to examine clustering between groups. Finally, bacterial diversity was calculated using the Shannon–Wiener Diversity Index.

#### Quantitative PCR analysis of bacteria

Stool, colonic mucus and small intestinal mucus DNA was used as a template for a quantitative polymerase chain reaction (qPCR) performed using a Fluidigm BioMark HD System with 48:48 IFC (Fluidigm, South San Francisco, CA) according to the manufacturer's instructions. Primers used are illustrated in Table 1. Universal primers for the 16S rRNA gene were used as a housekeeping control. Data were analysed using Fluidigm Real‐time PCR Analysis Software V4.3.1 (Fluidigm) and results were calculated using the ∆∆CT method. Cycling steps for all primers were set according to the manufacturer's instructions for a 48 × 48 chip: denaturing at 95° for 60 seconds, followed by 30 cycles of 96° for 5 seconds and 60° for 20 seconds.

#### Statistical analysis

All statistical analysis was performed using either graphpad prism 7 (GraphPad Software, La Jolla, CA) or R. Student's *t‐*test was used to compare crypt and villus length, muscle wall thickness, goblet cell number, inner mucus thickness and bacterial localization between genotypes in male mice. Permutational multivariate analysis of variance (permanova) was used to calculate differences in species presence between samples using the ‘adonis’ function in the vegan R package.^13^ Relative expression of quantified bacteria was compared between niche and genotype using two‐way analysis of variance (anova) with a Tukey's *post hoc* test. Additionally, when comparing young and aged female mice, two‐way anova with a Tukey's *post hoc* test was used to compare crypt and villus length, muscle wall thickness, goblet cell number, inner mucus thickness and bacterial localization between genotypes. Size of the experimental and control groups was chosen *a priori* to focus on biologically significant differences – power analyses in consultation with in‐house statisticians and extensive experience with these models suggested that effects of 30–50% magnitude would be of biological interest and the number of mice chosen per treatment gave an 80% chance of resolving such effects as statistically significant (*P *<* *0·05).

## Results

### Absence of eosinophils does not affect colonic or small intestinal morphology in young mice

Our overall aim was to investigate how the absence of eosinophils could influence the gut microbiota. However, we first needed to establish whether there were any changes to overall gut architecture in eosinophil‐deficient mice, specifically changes in intestinal crypt length and muscle wall thickness.

**Figure 1:**
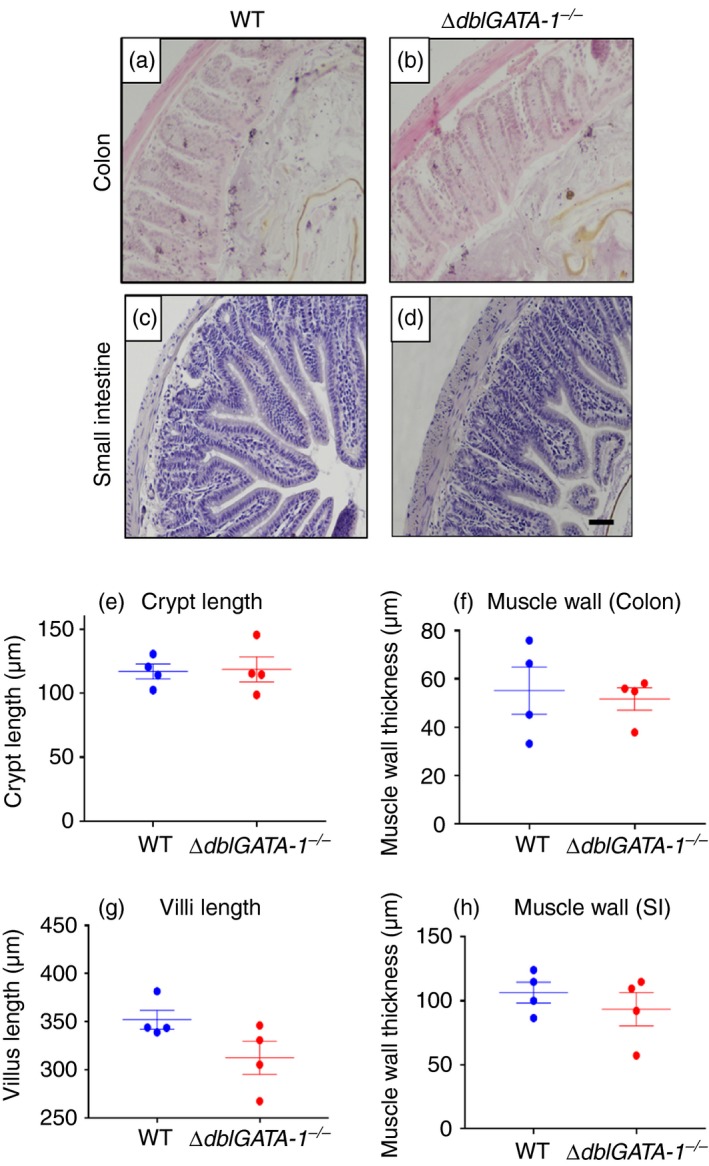
**Loss of eosinophils does not alter gut architecture.** Colonic and small intestinal tissue sections from 12‐week‐old male, C57BL/6 background wild‐type (WT) and eosinophil‐deficient (*∆dblGATA‐1* ^*−/−*^) littermate mice, were stained with haematoxylin & eosin to observe the gut morphology. Representative images were taken for the colon of WT mice (a) and *∆dblGATA‐1* ^*−/−*^ mice (b), and the small intestine of WT (c) and *∆dblGATA‐1* ^*−/−*^ mice (d). Colonic crypt length (e) and muscle wall thickness (f) and small intestinal villus length (g) and muscle wall thickness (h) were measured. Data are shown as mean ± standard error of the mean (SEM). *n* = 4 for all groups. Scale bar = 50 μm.

Haematoxylin & eosin staining revealed that there were no significant differences in either crypt length or muscle wall thickness of young male mice (12 weeks of age, Fig. 1). Additionally, we investigated the morphology of the small intestine and found no significant differences in villus length or muscle wall thickness between genotypes (Fig. 1). Hence, the absence of eosinophils did not alter gross gut morphology in male *∆dblGATA‐1* ^*−/−*^ mice.

When female mice were investigated, there were also no significant differences in muscle wall thickness between Het and *∆dblGATA‐1* ^*−/−*^ mice, in 12‐week‐old mice (young) or mice aged 1 year (aged) (see Supplementary material, Fig. S2). Young *∆dblGATA‐1* ^*−/−*^ mice tended to have longer colonic crypts than their Het counterparts (two‐way anova with Tukey's *post hoc* test: *P *=* *0·011), but this was restored to levels akin to Het in older mice (see Supplementary material, Fig. S1). There were no significant morphological differences in the small intestine between Het and *∆dblGATA‐1* ^*−/−*^ mice of any age (see Supplementary material, Fig. S3).

In addition to investigating the local impact on gut morphology, we also examined the more systemic impact on host function. Eosinophils have been shown to promote glucose homeostasis in the context of a high‐fat diet.^14^, ^15^ Under naive conditions, we did not find any differences in glucose tolerance when comparing our young Het versus young *∆dblGATA‐1* ^*−/−*^ mice or aged Het versus aged *∆dblGATA‐1* ^*−/−*^ mice (see Supplementary material, Fig. S4). Our previous work suggested that eosinophils had a mild impact on blood pressure,^16^ although no significant genotypic differences were identified in the present study (see Supplementary material Fig. S5).

### Mucus barrier integrity

**Figure 2:**
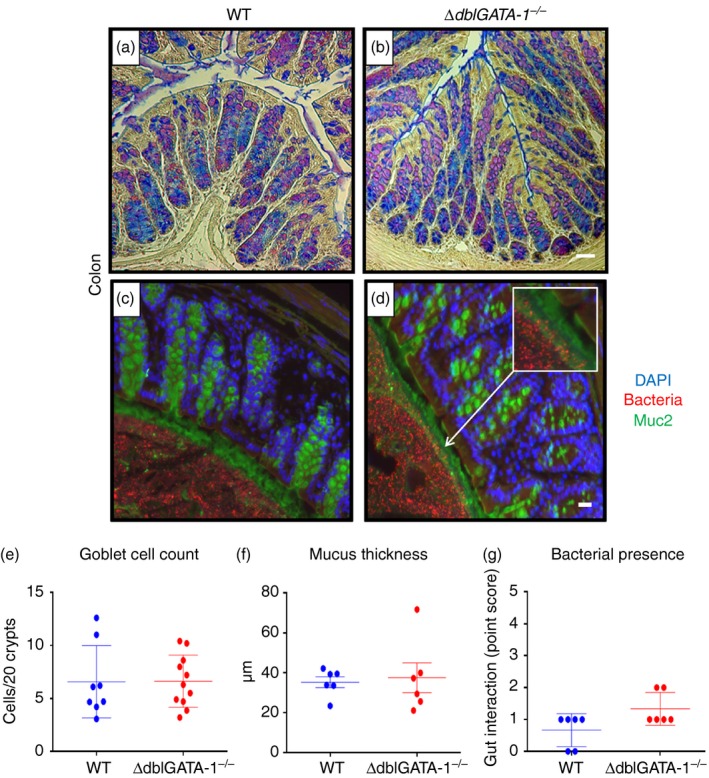
Mucus barrier integrity in *ΔdblGATA‐1* ^*−/−*^ mice. Colonic tissue sections were taken from 12‐week‐old male, C57BL/6 background wild‐type (WT) and eosinophil‐deficient (*ΔdblGATA‐1* ^*−/−*^) littermate mice. Goblet cells were stained using periodic acid, Alcian blue and Schiff's reagent and representative images for WT mice (a) and *∆dblGATA‐1* ^*−/−*^ mice (b) are displayed. Sections were also stained with a fluorescent DNA probe specific for the 16S rRNA gene to identify bacteria (red), a Muc2 antibody (green) to identify mucus and counterstained with DAPI (blue). Representative images for WT (c) and *∆dblGATA‐1* ^*−/−*^ mice (d) are displayed. Goblet cells (e), inner mucus thickness (f) and bacterial localization (g) were measured. Bacteria were scored based on their location within the gut: 0 = bacteria in the lumen and outer mucus layer, 1 = bacteria in the inner mucus layer, 2 = bacteria in contact with the epithelium, 3 = bacteria in the crypts, 4 = bacteria in the lamina propria. Data shown as mean ± SEM. *n* = 6 (WT) and 11 (*∆dblGATA‐1* ^*−/−*^). Scale bars = 50 μm.

Eosinophils may play a role in the regulation of the mucus barrier^5^ and so we quantified the number of goblet cells and the thickness of the colonic mucus layer (Fig. 2). Goblet cell numbers in the colon were equivalent between genotypes (Fig. 2e). There were also no significant differences in small intestinal goblet cell number (see Supplementary material, Fig. S6). We also investigated the thickness of the inner mucus layer and saw a similar inner mucus thickness between genotypes in male mice (Fig. 2f). Goblet cell numbers were similar between groups in female mice (see Supplementary material, Figs S7 and S8). However, younger, female eosinophil‐deficient mice had more variable mucus thickness when compared with Het mice, which was significantly different between genotypes (two‐way anova with Tukey's *post hoc* test: *P *=* *0·04; see Supplementary material, Fig. S9).

Irrespective of mucus thickness, the quality of the mucus barrier may be altered such that bacterial localization could be affected. We therefore stained colon sections with a fluorescent universal probe for bacteria, and a Muc2 antibody (Fig. 2). Sections were scored based on how far bacteria had travelled from the lumen to the lamina propria. Two mice that lacked eosinophils had evidence of increased bacterial localization further into the gut; however, most of the *∆dblGATA‐1* ^*−/−*^ mice were equivalent to their WT counterparts (Fig. 2g). Furthermore, bacterial localization was similar between WT and *∆dblGATA‐1* ^*−/−*^ female mice irrespective of age (see Supplementary material, Fig. S9).

**Figure 3:**
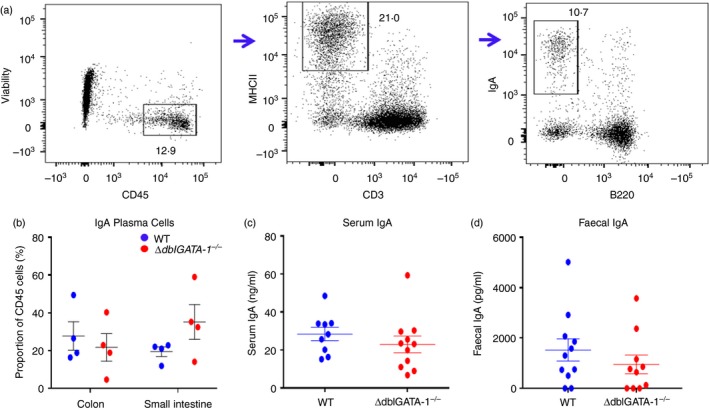
Loss of eosinophils does not impact IgA. Cells isolated from the colon and small intestine, from C57BL/6 background, male wild‐type (WT) and *∆dblGATA‐1* ^*−/−*^ littermate mice were gated to calculate the frequency of IgA^+^ plasma cells, gated as CD45^+^, MHCII ^+^, CD3^−^, IgA^+^ and B220^−^ (a). Numbers of IgA^+^ cells were reported as the total proportion of live CD45^+^ cells (b, *n* = 4). Serum (c, *n* = 9 WT and 11 *∆dblGATA‐1* ^*−/−*^) and stool homogenates (d, *n* = 11 WT and *n* = 10 *∆dblGATA‐1* ^*−/−*^) were analysed via ELISA to determine secreted levels of IgA. Data shown as mean ± standard error of the mean (SEM).

As IgA is a key component of the mucus barrier, we determined whether eosinophils play a role in the regulation of IgA‐secreting plasma cells (Fig. 3). Flow cytometry was used to define plasma cells which were gated as CD45^+^, MHCII^+^, CD3^−^, IgA^+^ and B220^−^. Analysis of colonic and small intestinal cells isolated from male, WT and *∆dblGATA‐1* ^*−/−*^ mice revealed no striking differences in the number of IgA‐expressing cells between genotypes (Fig. 3a,b). This was concordant with no differences in serum or faecal IgA levels between young male WT and *∆dblGATA‐1* ^*−/−*^ mice (Fig. 3c,d). However, in female mice there was a trend towards higher levels of serum IgA in *∆dblGATA‐1* ^*−/−*^ mice compared with Het, with significant differences in younger *∆dblGATA‐1* ^*−/−*^ mice compared with Het (two‐way anova with Tukey's *post hoc* test: *P *=* *0·002) (see Supplementary material, Fig. S10).

### Eosinophils influence microbial diversity

**Figure 4:**
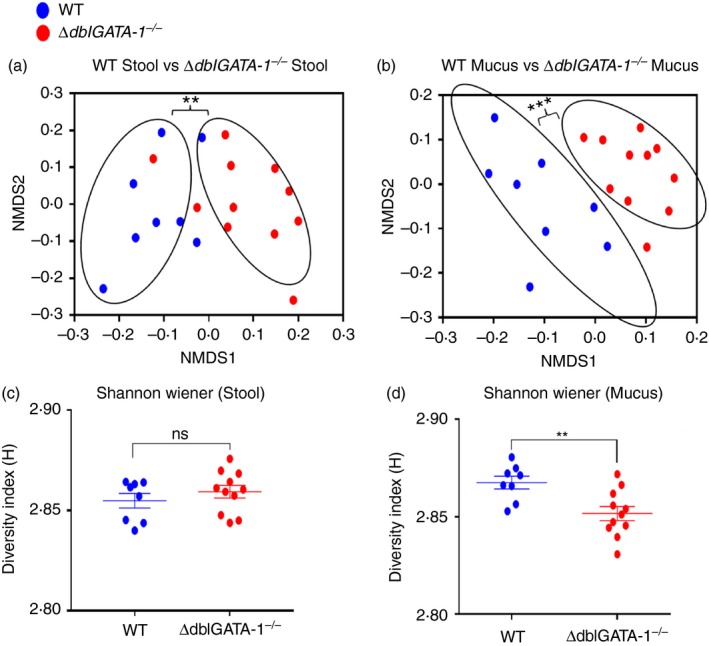
Absence of eosinophils leads to significantly altered gut microbiota. Differences in bacterial species composition and diversity between the stools and mucus of C57BL/6 background, male wild‐type (WT) and *ΔdblGATA‐1* ^*−/−*^ littermate mice, were analysed by denaturing gel gradient electrophoresis. Differences in the bacterial communities were plotted using non‐metric multidimensional scaling, for the stool (a) and mucus (b) communities. Rings indicate a significant difference between the bacterial communities of the respective treatment groups, as determined by permutational multivariate analysis of variance (**P < 0·01, ***P < 0·001). Subsequent diversity analysis was then performed on stool (c) and mucus (d). Data shown as mean ± standard error of the mean (SEM). *n* = 8 (WT) and 11 (*∆dblGATA‐1^−/−^*).

We next investigated whether the bacterial communities themselves could be affected by eosinophil deficiency. We used a gel‐fingerprinting technique (denaturing gradient gel electrophoresis; DGGE) to provide an overview of the bacterial communities in our WT and *∆dblGATA‐1* ^*−/−*^ mice (Fig. 4). Although the stool communities were significantly different between genotypes (permanova:* P *=* *0·001), the mucus microbiota had a clearer and more significant separation between genotypes (permanova:* P *=* *0·00003). Additional plots showing the samples coloured by cage and mother revealed a limited cage effect influencing the stool and mucus microbiome of the male mice (see Supplementary material, Fig. S11). *α*‐Diversity, a measure of the range of different bacteria within samples, revealed that eosinophil‐deficient mice had significantly lower diversity in the mucus compared with WT mucus (two‐way anova with Tukey's *post hoc* test: *P *=* *0·0018). In contrast, there was no difference in stool *α*‐diversity between genotypes.

In aged female mice, there was no clear difference between Het and *∆dblGATA‐1* ^*−/−*^ stool although the stool microbiota was significantly different when comparing young and aged female *∆dblGATA‐1* ^*−/−*^ mice (permanova:* P *=* *0·004) (see Supplementary material, Fig. S12). We also saw no differences in stool diversity between Het and *∆dblGATA‐1* ^*−/−*^ mice (see Supplementary material, Fig. S12). When the mucus was examined in female mice, we saw significant differences between young Het and young *∆dblGATA‐1* ^*−/−*^ mice (permanova:* P *=* *0·02) but not the older mice. However, there was an age effect with young and aged Het mice and young and aged *∆dblGATA‐1* ^*−/−*^ mice having significantly separated bacterial fingerprints (permanova:* P *=* *0·01 and *P *=* *0·004, respectively) (see Supplementary material, Fig. S13). Additional plots showing the samples coloured by cage and mother reveal a strong cage effect influencing the stool and mucus microbiome of the female mice (see Supplementary material, Fig. S14).

We also saw a significant reduction in mucus diversity in aged relative to young *∆dblGATA‐1* ^*−/−*^ mice. However, we did not see a reduction in mucus diversity with age in Het genotypes. Taken together, the data from male and female mice would suggest that the lack of eosinophils does impact on the microbiome with a stronger influence on the mucus microbiota compared with the stool communities.

**Figure 5:**
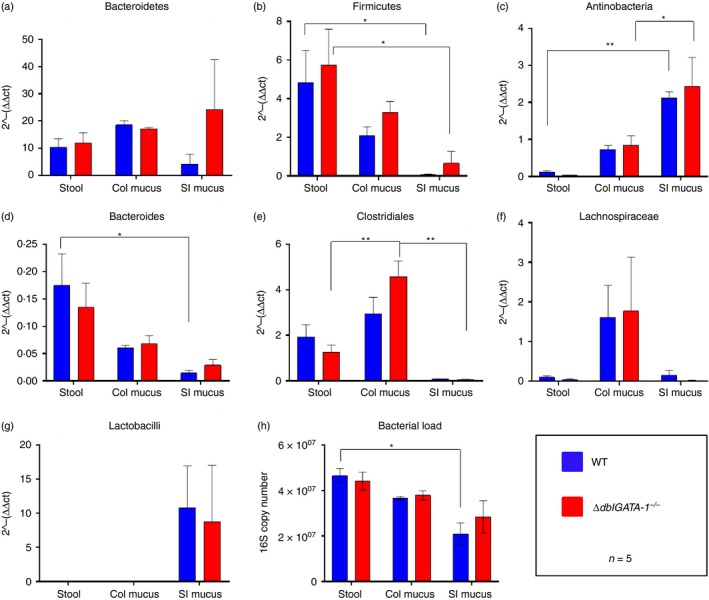
Bacterial communities significantly different between microbial niches. Quantitative PCR was used to assess expression of gut bacteria in C57BL/6 background, male wild‐type (WT) and *ΔdblGATA‐1* ^*−/−*^ littermate mice, in stool, colonic (col) mucus and small intestinal (SI) mucus. The relative expression of Bacteriodetes (a), Firmicutes (b), Actinobacteria (c), Bacteroides (d), Clostridiales (e), Lachnospiraceae and Ruminococcaceae (f), and Lactobacilli (g) are illustrated. Overall bacterial load is also displayed (h). Asterisks represent significant as determined by two‐way analysis of variance with a Tukey's *post hoc* test (*P < 0·05, **P < 0·01).Data shown as mean ± standard error of the mean (SEM). *n* = 5 for all groups.

We then quantified the differences in the microbiota via qPCR (Fig. 5). A panel of the most common bacterial phyla, orders, families and species that comprise the gut microbiota was selected. Unexpectedly, we saw no significant differences in overall microbial burden or in the levels of common gut phyla, orders and species between genotype. However, there were striking differences in the bacteria among microbial niches, with stool versus colonic mucus or small intestinal mucus containing significantly different bacteria. For example, at the phylum level in WT mice, we saw a significant reduction in Firmicutes (two‐way anova with Tukey's *post hoc* test: *P *=* *0·04) and a significant increase in small intestinal Actinobacteria (two‐way anova with Tukey's *post hoc* test: *P *=* *0·004) when comparing stool and small intestinal mucus. In *∆dblGATA‐1* ^*−/−*^ mice, we saw differences between the stool and small intestinal mucus, with a significant reduction in Firmicutes (two‐way anova with Tukey's *post hoc* test: *P *=* *0·01) and a significant increase in Actinobacteria (two‐way anova with Tukey's *post hoc* test: *P *=* *0·0006) in the small intestine. We saw no differences in the level of the phylum Bacteroidetes. At the order level, we saw a significant reduction in Bacteroides when comparing WT stool and small intestinal mucus (two‐way anova with Tukey's *post hoc* test: *P *=* *0·01). In *∆dblGATA‐1* ^*−/−*^ mice, we saw a significant increase in Clostridiales when comparing stool and colonic mucus (two‐way anova with Tukey's *post hoc* test: *P *=* *0·0007) and there was a significant reduction in Clostridiales in the small intestinal mucus compared with the colonic mucus (two‐way anova with Tukey's *post hoc* test: *P *=* *0·0001). At the family and genus level, we saw no significant differences in Lachnospiraceae, lactobacilli, Ruminococcaceae and segmented filamentous bacteria (SFB) (see Supplementary material, Fig. S15).

## Discussion

Eosinophils have previously been implicated in harmful inflammatory contexts such as allergy.^17^ However, more recent studies suggest that eosinophils play an active role in the maintenance of gut homeostasis, for example, in the regulation of IgA‐secreting plasma cells^4^, ^5^ and the mucus barrier. However, we found no differences in IgA levels when comparing young WT and eosinophil‐deficient mice, but showed a significant increase in IgA cells in older eosinophil‐deficient mice compared with younger mice. Previous studies have shown conflicting results with regards to the impact of eosinophils on IgA plasma cells with data implicating both positive and negative effects.^4^, ^5^, ^18^ One difference between our present study and the aforementioned studies is strain, where we use C57BL/6 mice as opposed to mice on a BALB/c background. It was previously shown that BALB/c mice natively have significantly higher levels of IgA production compared with C57BL/6 mice.^19^ It is also possible that differences in IgA may be niche‐specific and become apparent under the context of infection or inflammation. Our previous work showed that IgA differences were most apparent in infection‐induced inflammation and only in the small intestine in *∆dblGATA‐1* ^*−/−*^ mice of a BALB/c background.^4^ There may also be an effect of sex influencing IgA levels, as within our study only young, female eosinophil‐deficient mice had significantly higher levels of IgA in the serum compared with Het. However, this could be a consequence of female control mice being heterozygous, as opposed to true wild‐types.

Given the location of eosinophils within the gut, there could be cross‐talk between eosinophils and intestinal epithelial cells that maintain gut homeostasis that does not involve IgA. Indeed, eosinophils have been shown to influence mucus thickness.^5^ However, in our hands we saw no changes in goblet cell numbers or mucus thickness. Physical changes in the properties of the mucus, for example glycosylation and viscosity, however, could still impact on the microbiome. Our finding that there was not consistent altered gut bacterial penetrance suggests that eosinophils did not impact on bacterial localization, at least in homeostasis. However, eosinophils could still directly impact on the make‐up of the microbiota. For example, eosinophils contain a variety of cytotoxic granules that can have a notable physiological impact on the host.^20^ Indeed eosinophils interact with the gut microbiota to limit *Clostridium difficile* infection in an interleukin‐25‐dependent manner.^21^ It was notable in our study that mucus‐resident bacteria were markedly different from the stool bacteria as revealed by qPCR, fingerprint profiling and *α*‐diversity analysis. Given our observation that there were no striking differences in IgA production, we would hypothesize that the impact on mucus‐resident bacteria is either through production of epithelium‐derived anti‐bacterial peptides or secretion of anti‐bacterial factors from the eosinophils themselves.

With regards to the microbiota, it is known that various factors such as strain and cage effects have a marked impact on its composition.^22^, ^23^ Therefore the genetic background of the *∆dblGATA‐1* ^*−/−*^ mice could affect how eosinophils shape the microbiota. Hence, it would be interesting to investigate the microbiota in *∆dblGATA‐1* ^*−/−*^ mice of different genetic backgrounds under different perturbations such as diet or infection to test whether this could influence the microbiota composition and potentially host‐health. However, cage effect has been reported to have a stronger impact on gut microbiota variation than genetics.^24^ The microbiota is hugely dependent on the mother, food and the environment in which the mouse is reared,^25^, ^26^ so robust studies into the microbiota should control for this environment. Many microbiota studies do not report the use of littermate controls or crucial information about animal housing that could influence results.^27^, ^28^ Although previous work^5^ showed that there was a significantly altered microbial community in the stools of *∆dblGATA‐1* ^*−/−*^ mice, littermate controls were not reported as being used. Importantly, we controlled for littermates and saw that although there was an effect on stool microbiota, the most striking differences were in the mucus‐resident bacteria of littermate controls. Indeed, we demonstrated a reduction in mucus‐resident *α*‐diversity with no changes to stool *α*‐diversity in mice that lacked eosinophils.

Reduced gut microbial diversity is often associated with diseases such as inflammatory bowel disease^29^ and we have previously showed that changes in the mucus‐resident bacteria were implicated in the onset of gut inflammation.^7^ However, we saw no obvious impact on the health of *∆dblGATA‐1* ^*−/−*^ mice, based on their gut morphology. It is possible that changes in the mucus‐resident microbiota in this mouse model have no impact on host function on the parameters measured in this study. We assessed more systemic physiological effects, such as blood pressure and glucose tolerance, and saw no genotype differences. It might be interesting, however, to assess these populations in germ‐free mice. Another aspect that may be worth considering is that the mice in our study were not under any inflammatory challenge and it may be that changes in microbiota are more significant when there is an inflammatory insult. For instance, a high‐fat diet was shown to induce a reduction in eosinophils, associated with an increase in intestinal permeability.^3^ Although the exact mechanism for the increased permeability was not established, changes in the microbiota have been linked with altered gut permeability, where a high‐fat diet low in dietary fibre led to an increase in mucus‐degrading bacteria and degradation of the mucus barrier.^30^ Therefore, changes in the microbiota could be involved.

Although we saw overall differences in the gut microbiota by DGGE, our specific analysis by qPCR of common bacterial phyla, orders and families revealed no differences between genotypes in our mice. However, DGGE gives a broad microbial fingerprint for each sample, as opposed to qPCR, which focuses on specific bacteria. It is possible that bacteria that were significantly different between genotypes were not encompassed in our qPCR panel of common gut bacteria. Perhaps alternative mechanisms of eosinophil depletion, such as treatment with antibody against Siglec‐F, would highlight the impact of eosinophils on the microbiota more clearly. Alternatively, 16S rRNA sequencing could allow more quantitative characterization of where the differences in the microbiota identified by DGGE lie. Crucially further studies should consider niche as both our qPCR and DGGE analysis of stool, colonic and small intestinal mucus highlighted differences in the microbial population among those niches. It is known that the microbial composition between stool and mucus is significantly different.^7^, ^31^, ^32^ For example, one study showed that mucus samples had a greater relative abundance of Proteobacteria and Fusobacteria than stool, although the stool had a greater proportion of Firmicutes and Bacteroidetes.^32^ Taken together, these data emphasize the need to investigate the different microbial niches within the gut, to comprehensively explore the gut microbiota and the impact of genotype or environment.

Overall, we demonstrate that eosinophils did not influence IgA production, goblet cell number or mucus thickness. However, we show significant differences in the microbiota between niches. In addition, our DGGE analysis showed that eosinophils had a greater impact on the mucus‐resident bacterial communities compared with those in stool. These data reinforce the fact that a focus on stool is insufficient to capture the overall complexity of the gut microbiota and that eosinophils may play a more important role in regulating the mucus‐resident bacteria.

## Disclosures

The authors declare that they have no competing interests.

## Supporting information


**Figure S1.** Eosinophils in wild‐type and heterozygous small intestine.

**Figure S2.** Loss of eosinophils leads to altered gut morphology in old *∆dblGATA‐1* ^*−/−*^ mice.

**Figure S3.** Loss of eosinophils did not impact on morphological differences in small intestine structure.

**Figure S4.** Glucose tolerance unaffected by loss of eosinophils.

**Figure S5.** Blood pressure and pulse unaffected by loss of eosinophils.

**Figure S6.** Lack of eosinophils does not impact upon small intestinal goblet cells.

**Figure S7.** Loss of eosinophils does not impact upon goblet cell number in young or old mice.

**Figure S8.** Trend towards increased small intestinal goblet cells in *∆dblGATA‐1* ^*−/−*^ mice.

**Figure S9.** Inner mucus layer characterization.

**Figure S10.** Loss of eosinophils leads to increased serum IgA in younger female mice.

**Figure S11.** Limited cage effect impacting microbiome in stool and mucus samples from male wild‐type and *∆dblGATA‐1* ^*−/−*^ mice.

**Figure S12.** Differences in the bacterial communities and diversity in the stool of Heterozygous (Het) and eosinophil‐deficient (*∆dblGATA‐1* ^*−/−*^) mice.

**Figure S13.** Differences in the bacterial communities and diversity in the colonic mucus of Heterozygous (Het) and eosinophil‐deficient (*∆dblGATA‐1* ^*−/−*^) mice.

**Figure S14.** Strong cage effect impacting microbiome in stool and mucus samples from female wild‐type and *∆dblGATA‐1* ^*−/−*^ mice.

**Figure S15.** Expression of Enterobacteriaceae and segmented filamentous bacteria in wild‐type and *ΔdblGATA‐1* ^*−/−*^ mice.
