# Supplementary figures and images for: Gut eosinophils and their impact on the mucus‐resident microbiota
Source: Immunology. 2019 Sep 17;158(3):194–205. doi: 10.1111/imm.13110 (PMC6797872; doi:10.1111/imm.13110)

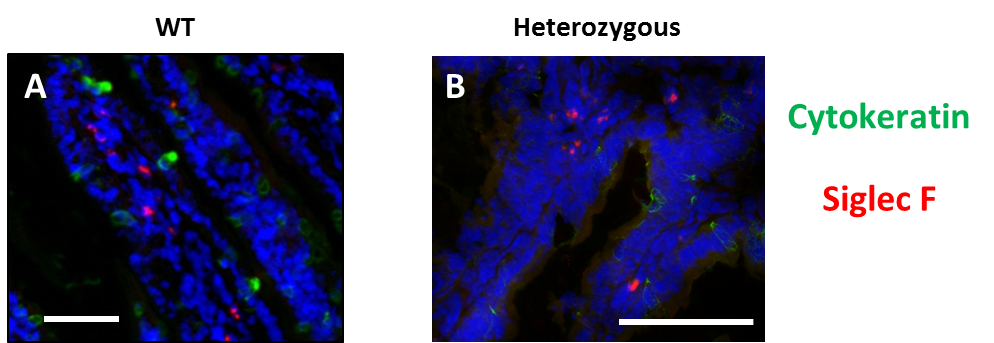

Supplement: Supplementary file 1 — Figure S1. Eosinophils in wild‐type and heterozygous small intestine. [file IMM-158-194-s001.tif]

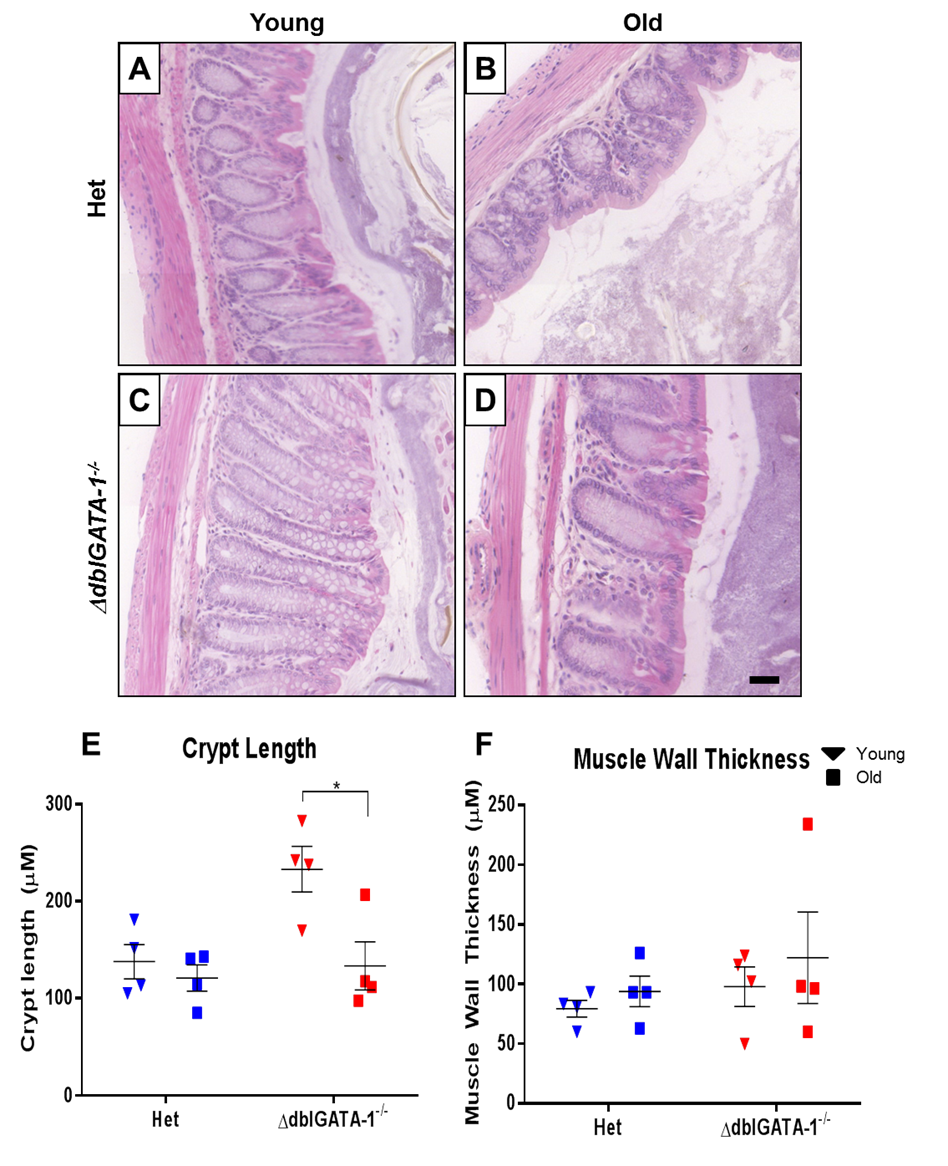

Supplement: Supplementary file 2 — Figure S2. Loss of eosinophils leads to altered gut morphology in old ∆dblGATA‐1 −/− mice. [file IMM-158-194-s002.tif]

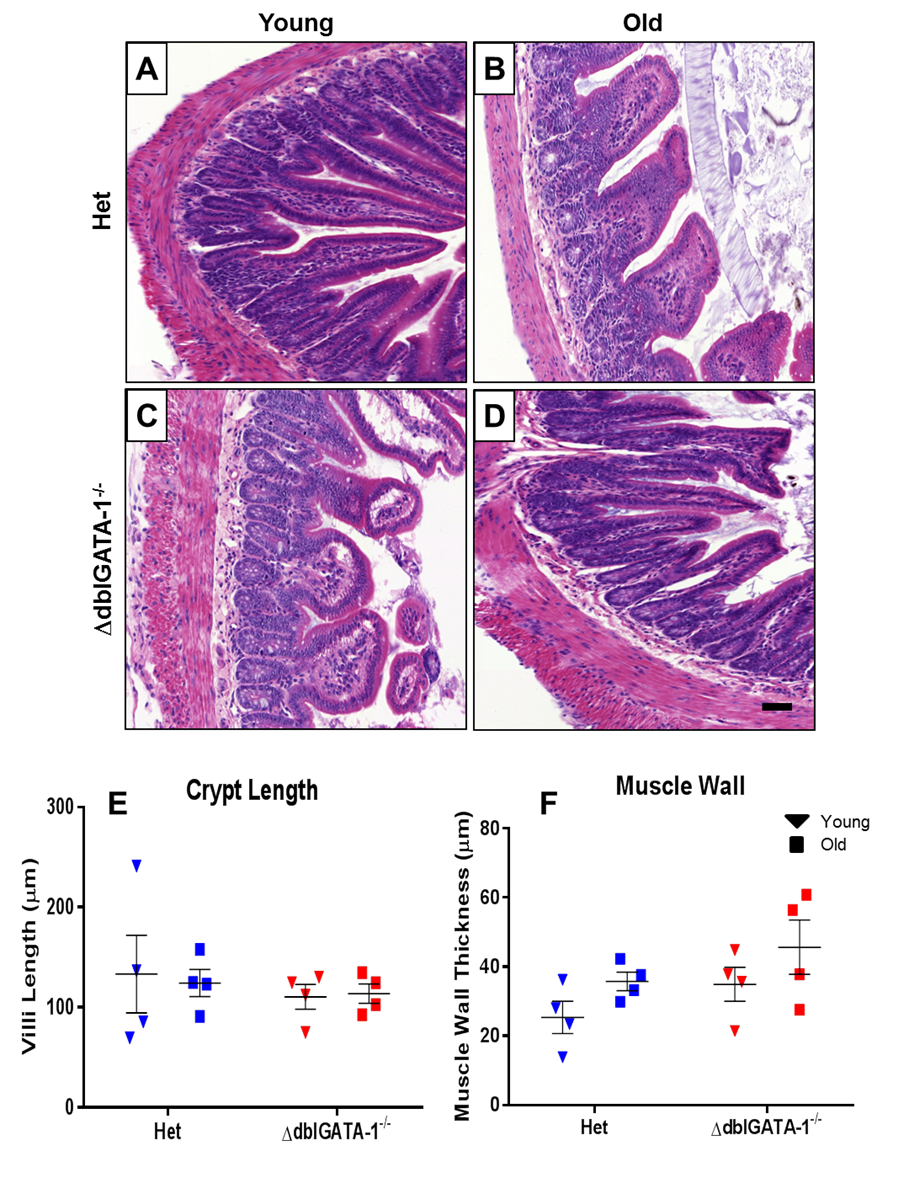

Supplement: Supplementary file 3 — Figure S3. Loss of eosinophils did not impact on morphological differences in small intestine structure. [file IMM-158-194-s003.tif]

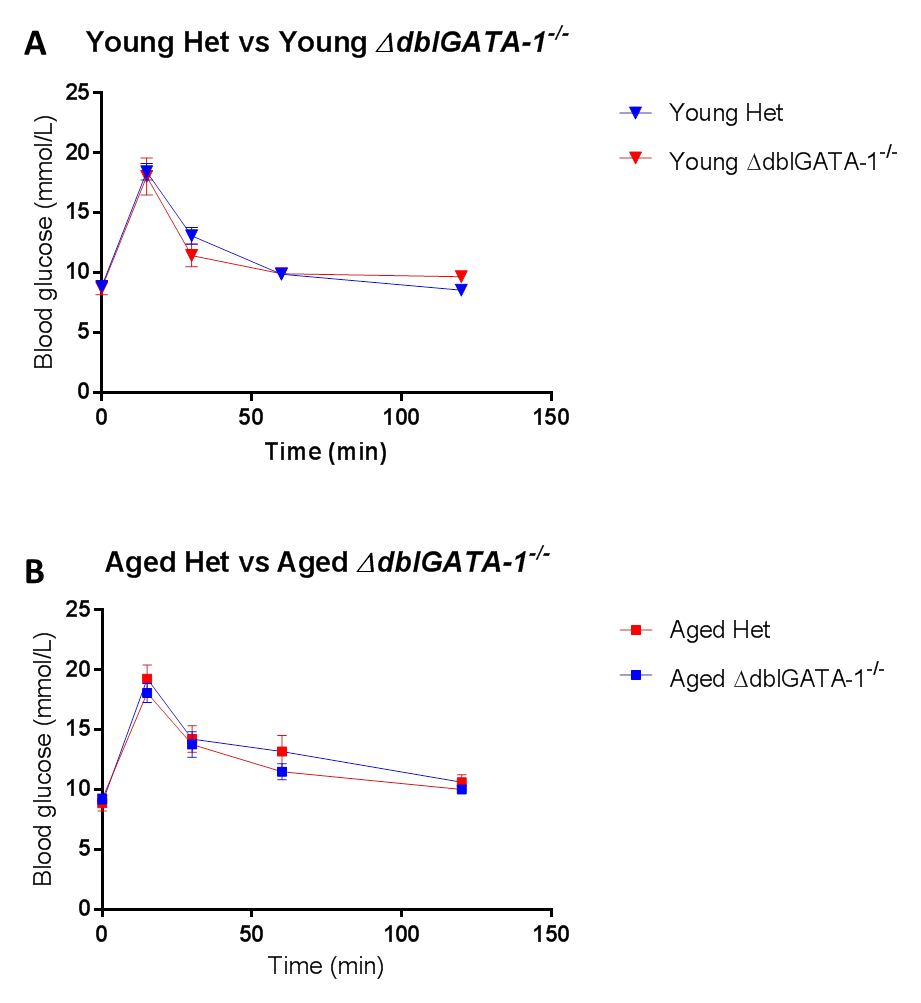

Supplement: Supplementary file 4 — Figure S4. Glucose tolerance unaffected by loss of eosinophils. [file IMM-158-194-s004.tif]

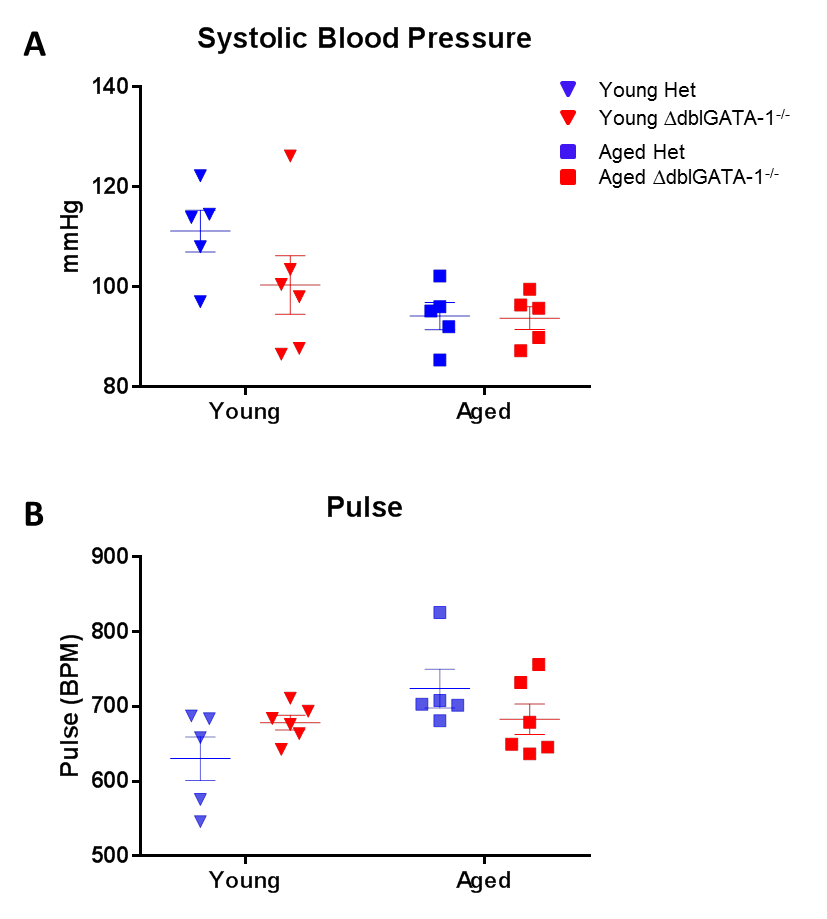

Supplement: Supplementary file 5 — Figure S5. Blood pressure and pulse unaffected by loss of eosinophils. [file IMM-158-194-s005.tif]

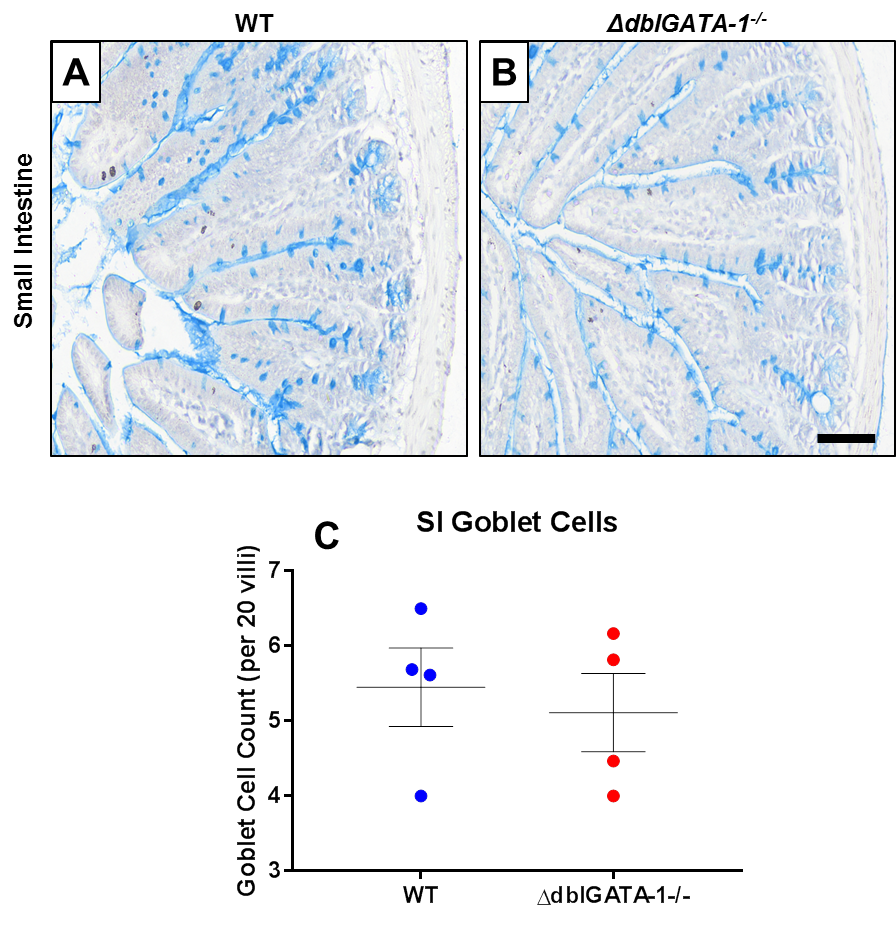

Supplement: Supplementary file 6 — Figure S6. Lack of eosinophils does not impact upon small intestinal goblet cells. [file IMM-158-194-s006.tif]

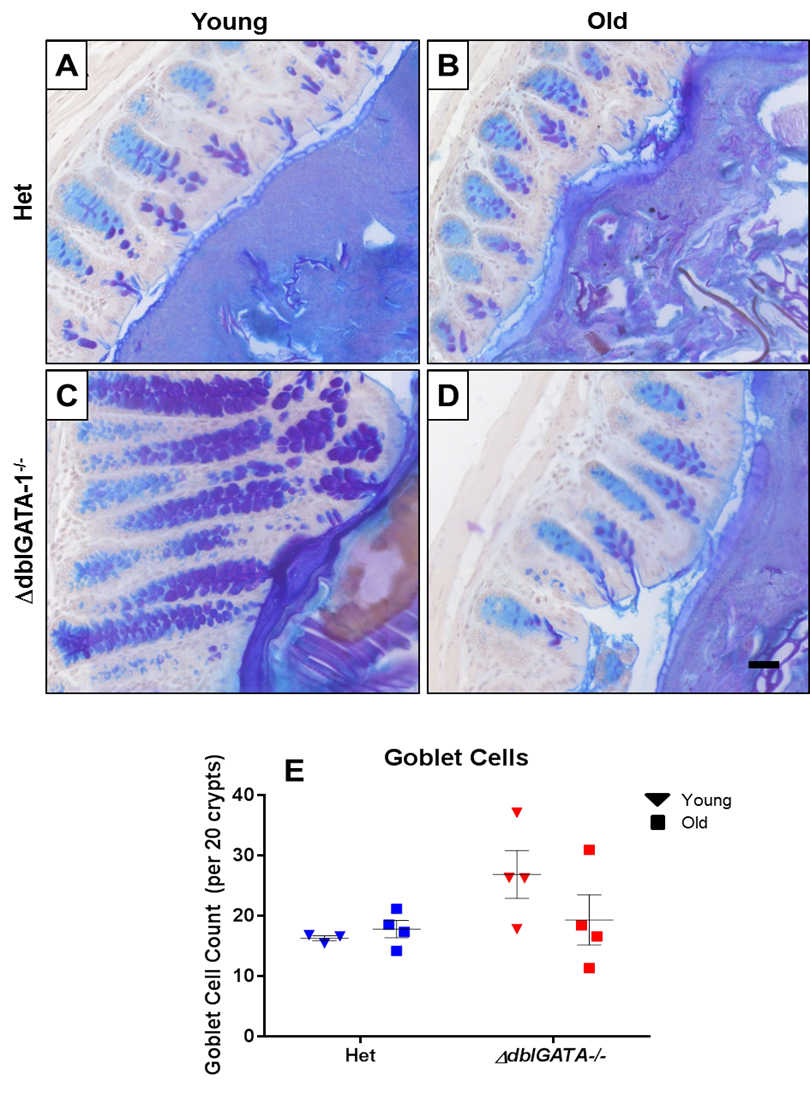

Supplement: Supplementary file 7 — Figure S7. Loss of eosinophils does not impact upon goblet cell number in young or old mice. [file IMM-158-194-s007.tif]

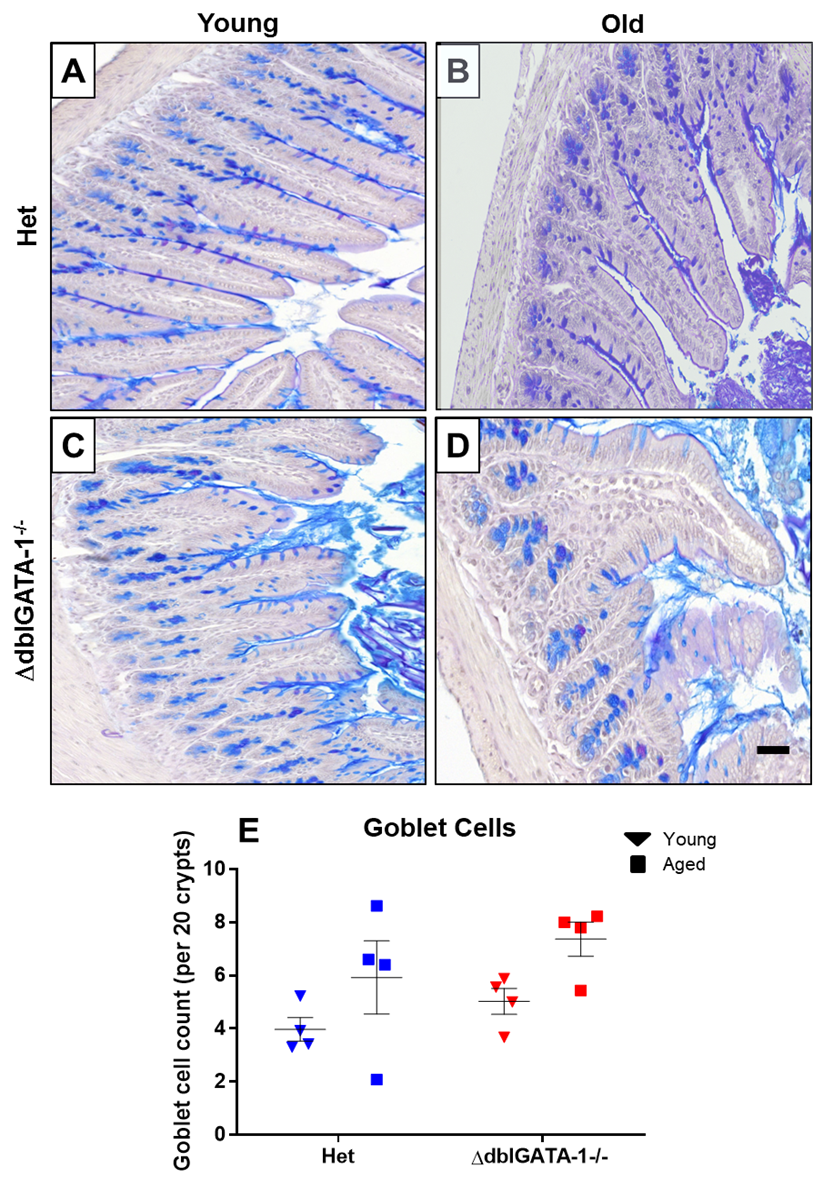

Supplement: Supplementary file 8 — Figure S8. Trend towards increased small intestinal goblet cells in ∆dblGATA‐1 −/− mice. [file IMM-158-194-s008.tif]

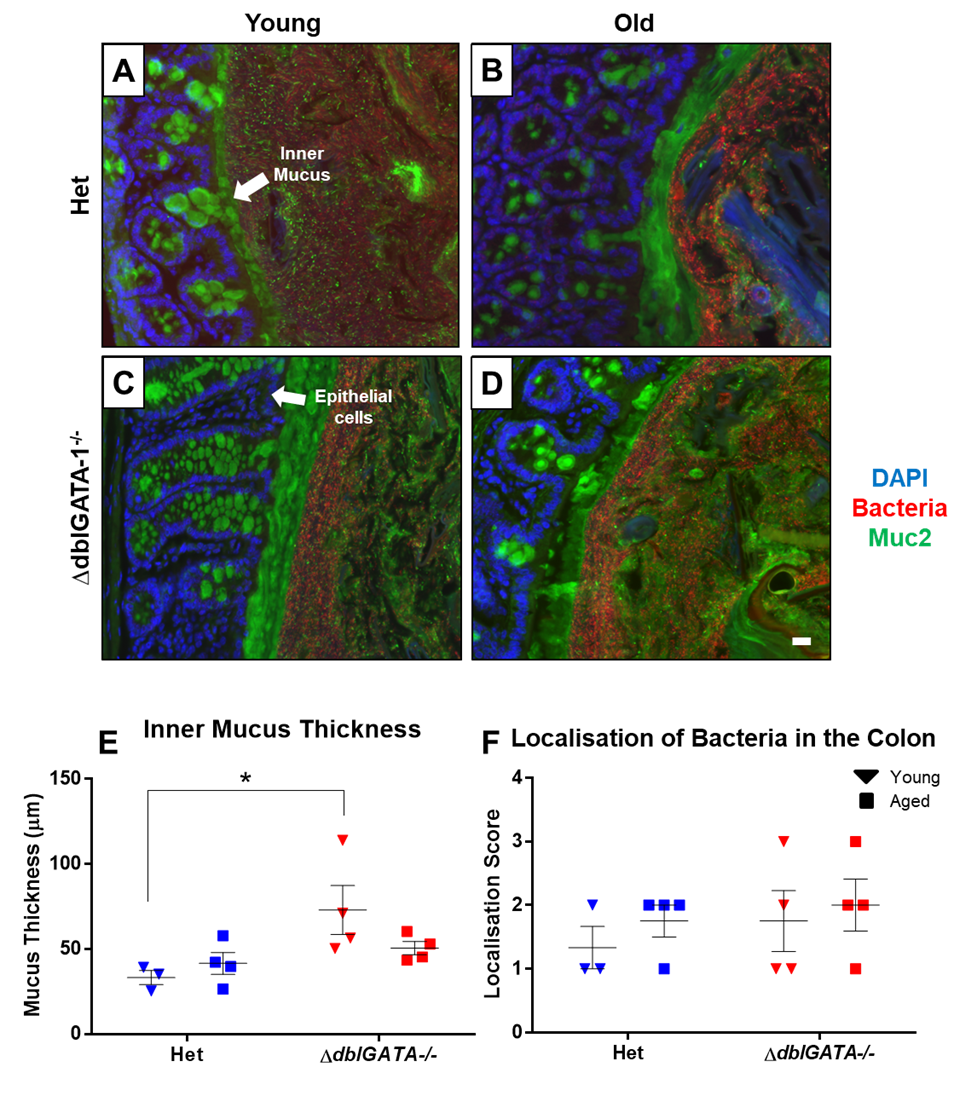

Supplement: Supplementary file 9 — Figure S9. Inner mucus layer characterization. [file IMM-158-194-s009.tif]

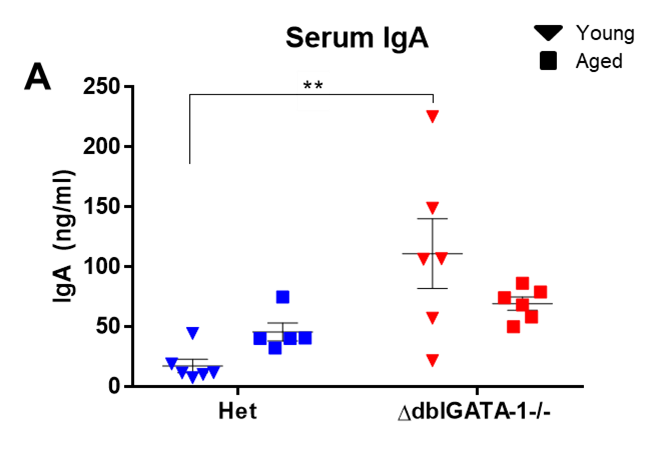

Supplement: Supplementary file 10 — Figure S10. Loss of eosinophils leads to increased serum IgA in younger female mice. [file IMM-158-194-s010.tif]

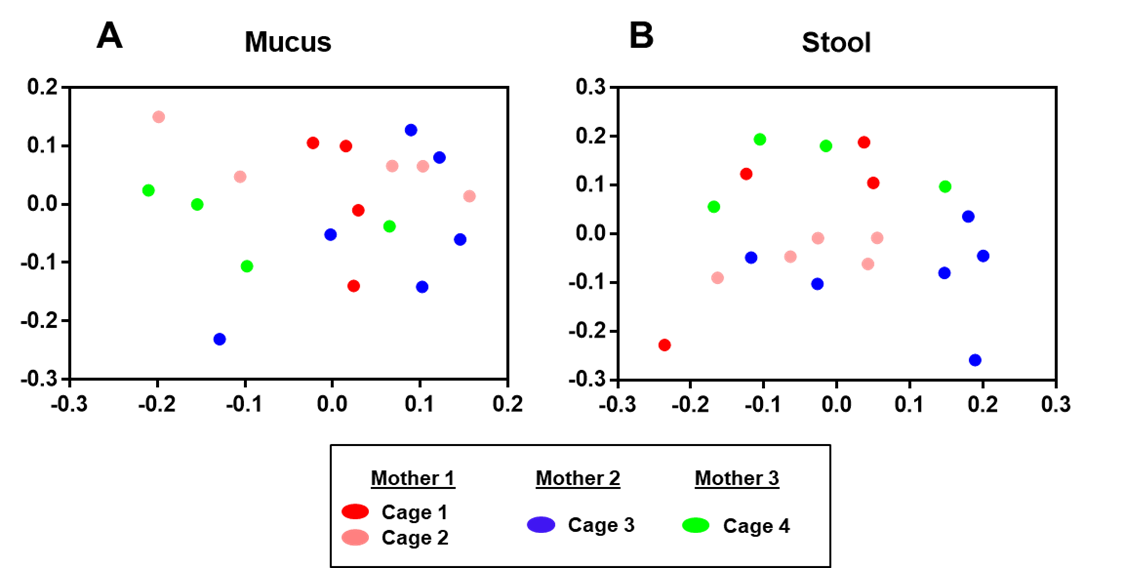

Supplement: Supplementary file 11 — Figure S11. Limited cage effect impacting microbiome in stool and mucus samples from male wild‐type and ∆dblGATA‐1 −/− mice. [file IMM-158-194-s011.tif]

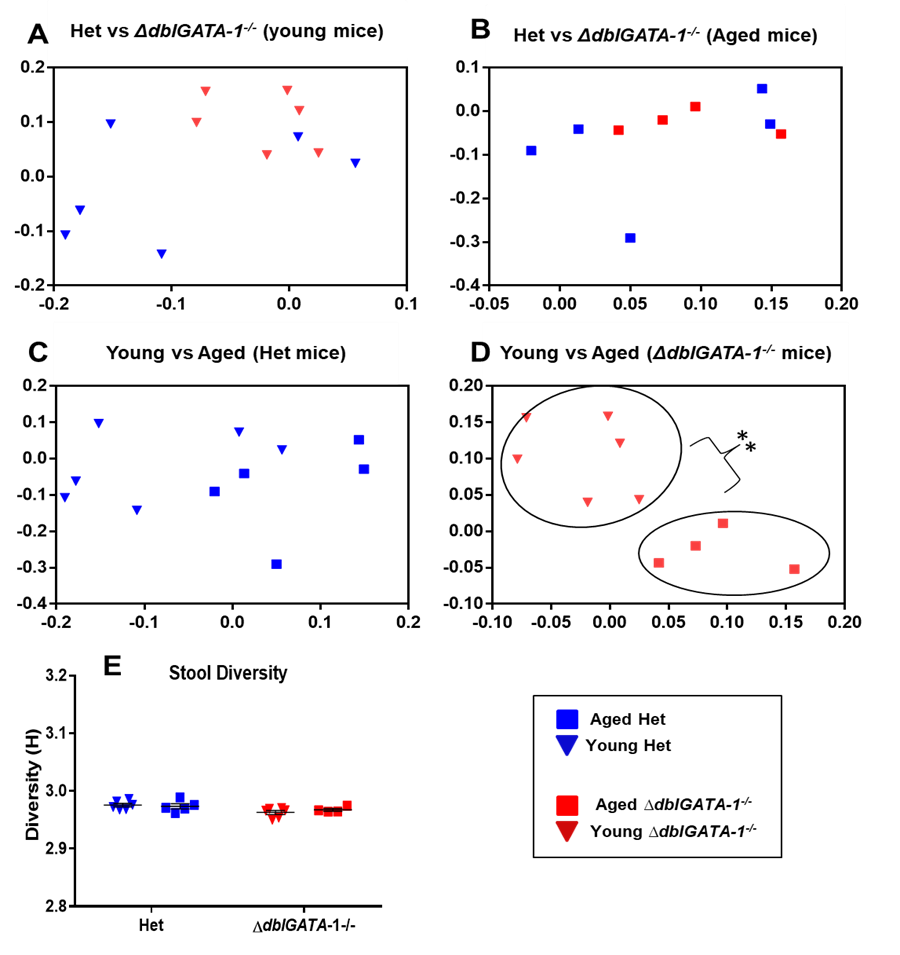

Supplement: Supplementary file 12 — Figure S12. Differences in the bacterial communities and diversity in the stool of Heterozygous (Het) and eosinophil‐deficient (∆dblGATA‐1 −/−) mice. [file IMM-158-194-s012.tif]

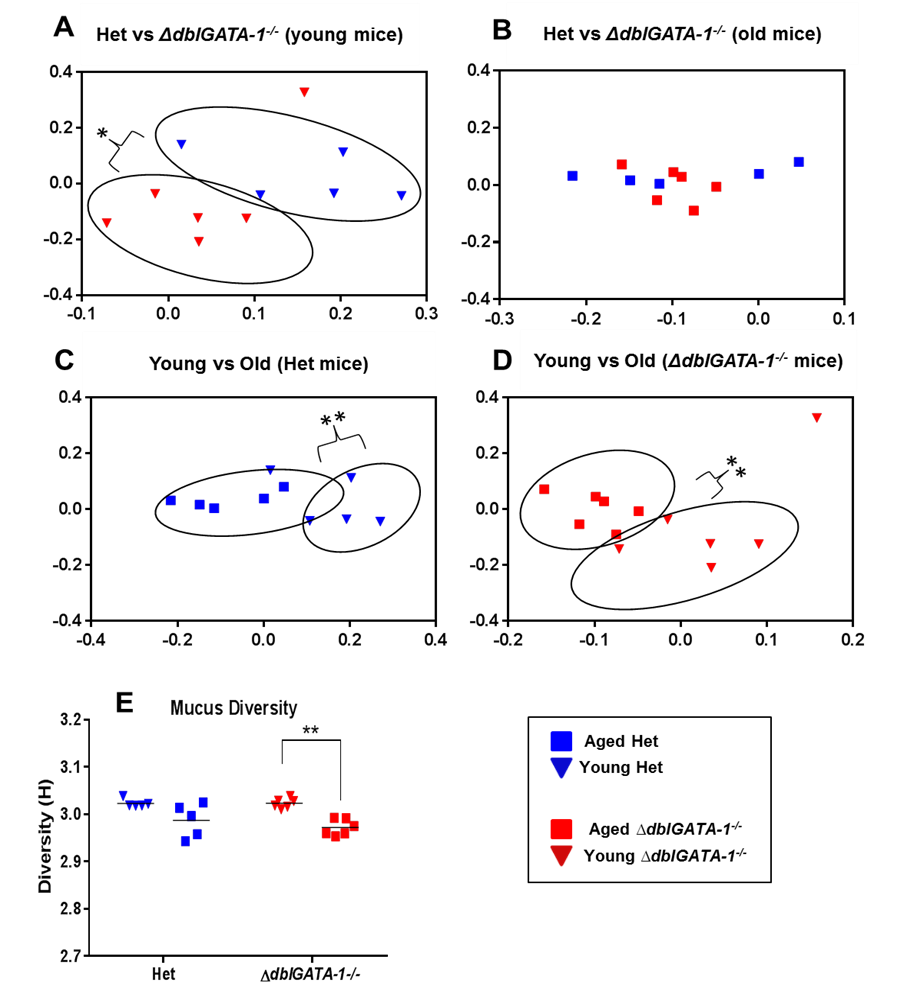

Supplement: Supplementary file 13 — Figure S13. Differences in the bacterial communities and diversity in the colonic mucus of Heterozygous (Het) and eosinophil‐deficient (∆dblGATA‐1 −/−) mice. [file IMM-158-194-s013.tif]

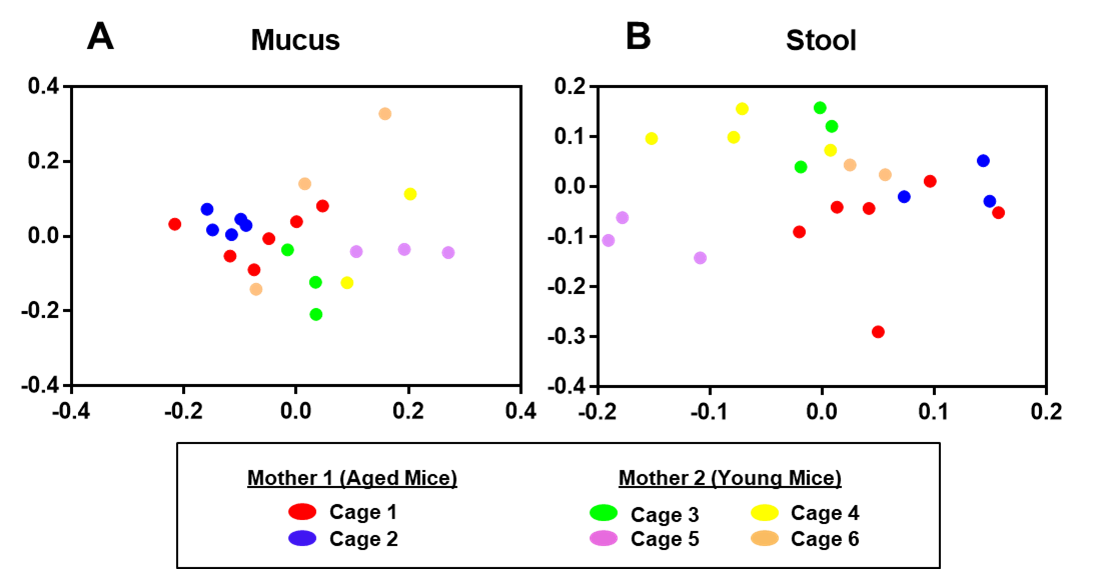

Supplement: Supplementary file 14 — Figure S14. Strong cage effect impacting microbiome in stool and mucus samples from female wild‐type and ∆dblGATA‐1 −/− mice. [file IMM-158-194-s014.tif]

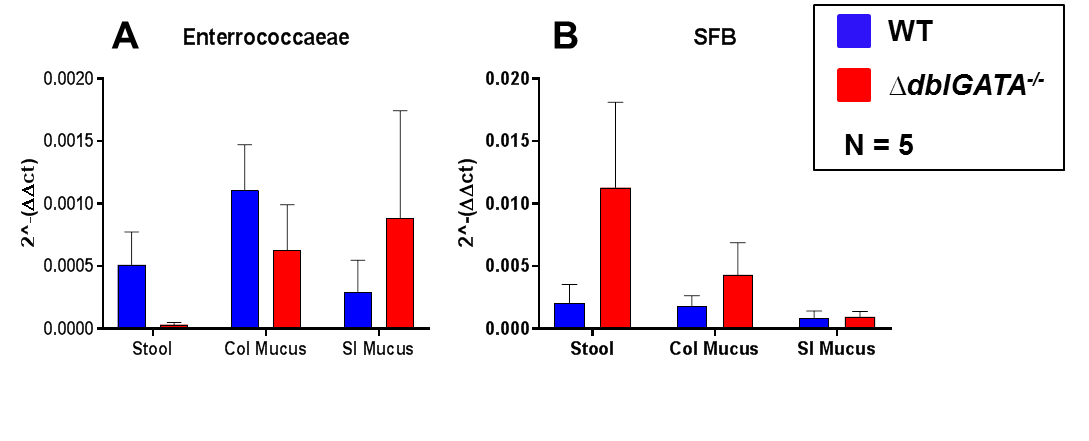

Supplement: Supplementary file 15 — Figure S15. Expression of Enterobacteriaceae and segmented filamentous bacteria in wild‐type and ΔdblGATA‐1 −/− mice. [file IMM-158-194-s015.tif]
