# Supplementary material for: Gut eosinophils and their impact on the mucus‐resident microbiota
Source: Immunology. 2019 Sep 17;158(3):194–205. doi: 10.1111/imm.13110 (PMC6797872; doi:10.1111/imm.13110)
Supplement: Supplementary file 16 [file IMM-158-194-s016.docx]

**Supplementary Figures**

**Supplementary Figure 1: Eosinophils in wildtype and heterozygous small intestine.** Small intestinal tissue sections were taken from 12 week old male, C57BL/6 background wildtype (WT) and female heterozygous (Het) mice. Eosinophils were stained using Siglec F (red) and epithelial cells were stained with cytokeratin (green). Representative images for WT mice (A) and Het mice (B) are displayed. Scale bars = 50µm.

**Supplementary Figure 2: Loss of eosinophils leads to altered gut morphology in old *∆dblGATA-1^-/-^* mice.** Colonic tissue sections from female 12 week (young) and 12 month (aged) old, C57BL/6 background heterozygous (Het) and eosinophil-deficient (*∆dblGATA-1^-/-^*) female mice, were stained with haematoxylin and eosin to observe the gut morphology. Representative images of young Het mice (A); old Het mice (B); young *∆dblGATA-1^-/-^* mice (C) and old *∆dblGATA-1^-/-^* mice (D). Colonic crypt length (E) and muscle wall thickness (F) were measured. Data is shown as mean +/- standard error of the mean (SEM). Asterisks represent significant as determined by 2 Way Analysis of Variance with a Tukey’s Post Hoc Test (p* = <0.05). n = 4 for all groups. Scale bar = 50μm.

**Supplementary Figure 3: Loss of eosinophils did not impact on morphological differences in small intestine structure.**  Small intestinal tissue sections from 12 week (young) and 12 month (aged) old, C57BL/6 background heterozygous (Het) and eosinophil-deficient (*∆dblGATA-1^-/-^*) female mice, were stained with haematoxylin and eosin. Representative images were taken from young mice (A) and old (B) Het mice; young (C) and old (D) *∆dblGATA-1^-/-^* mice and small intestinal crypt length (E) and muscle wall thickness (F) were measured. Data is shown as mean +/- standard error of the mean (SEM). Asterisks represent significant as determined by 2 Way Analysis of Variance with a Tukey’s Post Hoc Test (p* = <0.05). n = 4. Scale bar = 50μm.

**Supplementary Figure 4: Glucose tolerance unaffected by loss of eosinophils.** 12 week (young) and 12 month (aged) old, C57BL/6 background heterozygous (Het) and eosinophil-deficient (*∆dblGATA-1^-/-^*) female mice were fasted for 6 hours. Glucose concentrations were measured in tail vein samples at 0 min. Mice received an oral injection of 2 g/kg glucose and blood glucose was measured in tail vein blood after 15, 30, 60 and 120 min. Data shown as mean +/- SEM. n = 6 (Young Het mice, aged Het and (*∆dblGATA-1^-/-^*mice), n = 7 (young *∆dblGATA-1^-/-^* mice).

**Supplementary Figure 5: Blood pressure and pulse unaffected by loss of eosinophils.** 12 week (young) and 12 month (aged) old, C57BL/6 background heterozygous (Het) and eosinophil-deficient (*∆dblGATA-1^-/-^*) female mice were acclimatised to a blood pressure and pulse measuring procedure. Specifically, systolic blood pressure was assessed using a non-invasive blood pressure analyser with a specific mouse tail cuff adapter. On the day of assessment, the animals were placed in the room and allowed to acclimatise for 10 min. A total of 20 readings of systolic blood pressure (mmHg) (A) and heart rate (beats/min, bpm) (B) were taken and the average of the last 10 measurements calculated. Data shown as mean +/- SEM. n = 6 (Young Het mice, aged Het and *∆dblGATA-1^-/-^* mice), n = 7 (young *∆dblGATA-1^-/-^* mice).

**Supplementary Figure 6: Lack of eosinophils does not impact upon small intestinal goblet cells.** Small intestinal (SI) tissue sections were taken from 12-week old male, C57BL/6 background wildtype (WT) and eosinophil-deficient (*∆dblGATA-1^-/-^*) littermate mice. Goblet cells were stained using periodic acid, Alcian blue and Schiff’s reagent and representative images for WT mice (A) and *∆dblGATA-1^-/-^* mice (B) are displayed. Goblet cells were quantified (C). Data is shown as mean +/- standard error of the mean (SEM). n = 4 for all groups. Scale bar = 100μm.

**Supplementary Figure 7: Loss of eosinophils does not impact upon goblet cell number in young or old mice.**  Colonic tissue sections from 12 week (young) and 12 month (aged) old, C57BL/6 background heterozygous (Het) and eosinophil-deficient (*∆dblGATA-1^-/-^*) female mice, were stained for mucopolysaccharides using alcian blue, periodic acid and Schiff’s reagent. Representative images were taken for each treatment group: young Het mice (A, n = 3); old Het mice (B, n = 4); young *∆dblGATA-1^-/-^* mice (C, n = 4) and old *∆dblGATA-1^-/-^* mice (D, n = 4). Goblet cells were measured (E). Data is shown as mean +/- standard error of the mean (SEM). n = 3-4. Scale bar = 50μm.

**Supplementary Figure 8: Trend towards increased small intestinal goblet cells in *∆dblGATA-1^-/-^* mice.**  Small intestinal tissue were obtained, from 12 week (young) and 12 month (aged) old, C57BL/6 background heterozygous (Het) and eosinophil-deficient (*∆dblGATA-1^-/-^*) female mice. Sections were stained for mucopolysaccharides using alcian blue, periodic acid and Schiff’s reagent. Representative images were taken for each treatment group: young Het mice (A); old Het mice (B); young *∆dblGATA-1^-/-^* mice (C) and old *∆dblGATA-1^-/-^* mice (D). Goblet cells were measured (E). Data is shown as mean +/- standard error of the mean (SEM). n = 4 for all groups. Scale bar = 50μm.

**Supplementary Figure 9: Inner mucus layer characterisation.** Colonic tissue sections from 12 week (young) and 12 month (aged) old, C57BL/6 background heterozygous (Het) and eosinophil-deficient (*∆dblGATA-1^-/-^*) female mice were stained with a fluorescent DNA probe specific for the 16S rRNA gene to identify bacteria (red), Muc2 antibody (green) to identify mucus and counterstained with DAPI (blue). Representative images were taken for each treatment group: young Het mice (A, n = 3); old Het mice (B, n = 4), young *∆dblGATA-1^-/-^* mice (C, n = 4) and *∆dblGATA-1^-/-^* mice (D, n = 4). Inner mucus thickness (E) and bacterial localisation (F) were measured. Bacteria were scored based on their location within the gut: 0 = bacteria in the lumen and outer mucus layer, 1 = bacteria in the inner mucus layer, 2 = bacteria in contact with the epithelium, 3 = bacteria in the crypts, 4 = bacteria in the lamina propria. Data is shown as mean +/- standard error of the mean (SEM). Asterisks represent significant as determined by 2 Way Analysis of Variance with a Tukey’s Post Hoc Test (p* = <0.05). Scale bar = 50μm.

**Supplementary Figure 10: Loss of eosinophils leads to increased serum IgA in younger female mice.** Serum was analysed via ELISA to determine levels of IgA in 12 week old and 1 year old (aged) C57BL/6 background heterozygous (Het) and *∆dblGATA-1^-/-^* female mice. Data shown as mean +/- standard error of the mean (SEM). Asterisks represent significant as determined by 2 Way Analysis of Variance with a Tukey’s Post Hoc Test (p* = <0.05). n = 6 (young Het mice, young *∆dblGATA-1* mice and aged *∆dblGATA-1*mice ), n = 5 (aged Het mice).

**Supplementary Figure 11: Limited cage effect impacting microbiome in stool and mucus samples from male wildtype and *∆dblGATA-1^-/-^* mice.**  Differences in bacterial species composition and diversity between the gut mucus (A) and stools (B) of 12 week male C57BL/6 background wildtype and *∆dblGATA-1^-/-^* male mice, were analysed by denaturing gel-gradient electrophoresis. Differences in the bacterial communities were plotted using non-metric multidimensional scaling (NMDS). Data has been coloured by the cage that mice were housed in. Mice from cage 1 and 2 originate from Mother 1. Mice from cage 3 originate from Mother 2. Mice from cage 4 originate from Mother 3.

**Supplementary Figure 12: Differences in the bacterial communities and diversity in the stool of Heterozygous (Het) and eosinophil deficient (*∆dblGATA-1^-/-^*) mice.**  Differences in bacterial species composition and diversity between the stools of 12 week (young) and 12 month (aged) old, C57BL/6 background heterozygous (Het) and *∆dblGATA-1^-/-^* female mice, were analysed by denaturing gel-gradient electrophoresis. Differences in the bacterial communities were plotted using non-metric multidimensional scaling (NMDS) for Young Het mice versus young *∆dblGATA-1^-/-^* mice (A); Old Het mice versus old *∆dblGATA-1^-/-^* mice; Young Het mice versus old Het mice (C); young *∆dblGATA-1^-/-^* mice versus old *∆dblGATA-1^-/-^* mice (D). Rings indicate significant differences between the bacterial communities of the respective treatment groups, as determined by permutational multivariate analysis of variance (p** = <0.01). Subsequent diversity analysis was then performed on stools (E). Data is shown as mean +/- standard error of the mean (SEM). n = 4 (aged *∆dblGATA-1* mice), n = 5 (aged Het mice), n = 6 (young Het and young *∆dblGATA-1* mice).

**Supplementary Figure 13: Differences in the bacterial communities and diversity in the colonic mucus of Heterozygous (Het) and eosinophil deficient (*∆dblGATA-1^-/-^*) mice.** Differences in bacterial species composition and diversity between the colonic mucus of 12 week (young) and 12 month (aged), C57BL/6 background heterozygous (Het) and *∆dblGATA-1^-/-^* female mice, were analysed by denaturing gel-gradient electrophoresis. Differences in the bacterial communities were plotted using non-metric multidimensional scaling (NMDS) for Young Het mice versus young *∆dblGATA-1^-/-^* mice (A); Old Het mice versus old *∆dblGATA-1^-/-^* mice; Young Het mice versus old Het mice (C); young *∆dblGATA-1^-/-^* mice versus old *∆dblGATA-1^-/-^* mice (D). Rings indicate significant differences between the bacterial communities of the respective treatment groups, as determined by permutational multivariate analysis of variance (p** = <0.01). Subsequent diversity analysis was then performed on mucus (E). Data is shown as mean +/- standard error of the mean (SEM). n = 5 (young and aged Het mice), n = 6 (young and aged *∆dblGATA-1* mice).

**Supplementary Figure 14: Strong cage effect impacting microbiome in stool and mucus samples from female wildtype and *∆dblGATA-1^-/-^* mice.** Differences in bacterial species composition and diversity between the gut mucus (A) and stools (B) of 12 week (young) and 12 month (aged) old, C57BL/6 background heterozygous (Het) and *∆dblGATA-1^-/-^* female mice, were analysed by denaturing gel-gradient electrophoresis. Differences in the bacterial communities were plotted using non-metric multidimensional scaling (NMDS). Data has been coloured by the cage that mice were housed in. Mice from cage 1 and 2 originate from Mother 1. Mice from cages 3-6 originate from Mother 2.

**Supplementary Figure 15: Expression of Enterrococcaceae and SFB in WT and *ΔdblGATA-1^-/-^* mice**. Real-time quantitative PCR was used to assess expression of gut bacteria in 12 week old male WT and *ΔdblGATA-1^-/-^* mice. DNA was extracted from stool, colonic mucus and small intestinal (SI) mucus and used as a template for qPCR, using a 16S rRNA gene as a housekeeping control. The relative expression of Enterrococcaeae (A) and Segmented Filamentous Bacteria (SFB) are illustrated. Data shown as mean +/- standard error of the mean (SEM). n = 5 for all groups.
